# A 49-year-old with chest pain and collapse

**DOI:** 10.1136/heartjnl-2016-310923

**Published:** 2017-03-11

**Authors:** Alastair J Moss, Kelvin HH Lim, Alan G Japp

**Affiliations:** 1 Centre for Cardiovascular Science, University of Edinburgh, Edinburgh, UK; 2 Department of Cardiothoracic Surgery, Royal Infirmary of Edinburgh, Edinburgh, UK

**Keywords:** Cardiac computer tomographic (CT) imaging, Coronary artery disease

## Clinical introduction

**Figure 1:**
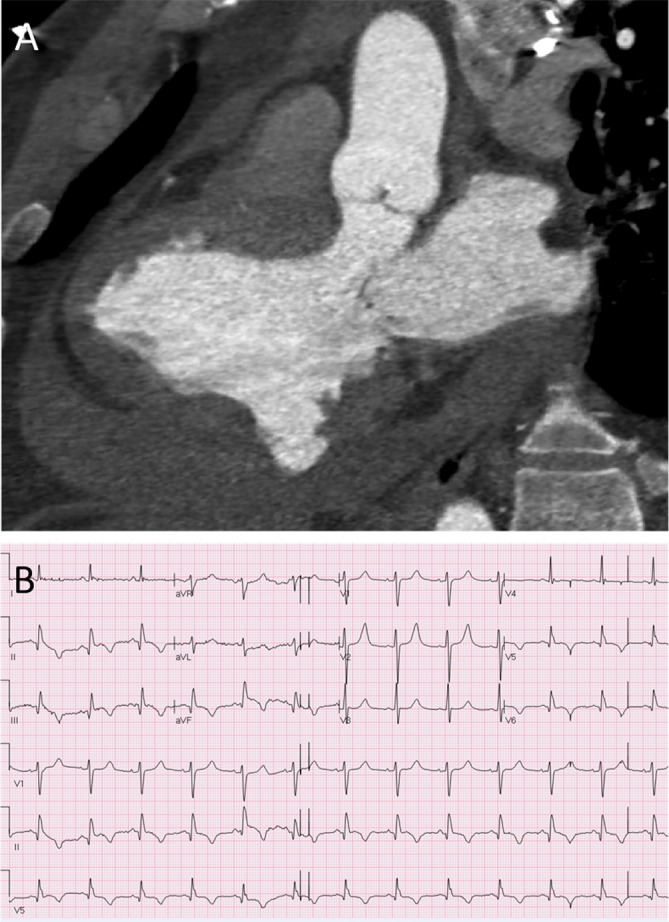
(A) CT angiogram with multiplanar reconstructions in three-chamber and axial views. (B) 12-lead ECG.

A 49-year-old man presented to the emergency department following sudden onset chest pain with collapse. He was refurbishing his home when he collapsed on the floor with chest and abdominal pain. He awoke 1 hour later and called the emergency services due to persisting chest discomfort that worsened with inspiration. On arrival in the emergency department, his pulse was thready (88/58 mm Hg) with pulsus paradoxus on inspiration. High-sensitivity troponin I was elevated at 325 ng/L (normal range 1–34 ng/L). Fluid resuscitation was administered and contrast-enhanced CT imaging was performed. (Figure 1).

## Question

Which of the following best explains this presentation?

A. Type A aortic intramural haematoma

B. Left ventricular diverticulum rupture

C. Malignant pericardial effusion

D. Left ventricular pseudoaneurysm

E. Blunt cardiac trauma

## Answer: D

The correct answer is left ventricular pseudoaneurysm. CT angiography demonstrates a large pericardial effusion with superior extension into the aortic and pulmonary recesses. The pericardium is not calcified and the pericardial fluid has a CT number of 43 Hounsfield units that suggests recent blood accumulation. There is discontinuity of the inferolateral wall with a filling defect containing iodinated contrast and no extravasation into the pericardial space. This is a contained rupture of the inferolateral left ventricular wall with thrombus formation preventing catastrophic exsanguination.

There is no intimal flap or aortic wall haematoma to indicate acute aortic syndrome. Left ventricular diverticula are outpouchings of myocardium usually found at the apex, which in combination with diaphragmatic and sternal defects constitute Cantrell’s syndrome.^1^ Blunt cardiac trauma often involves the right heart chambers due to their close proximity to the sternum in conjunction with deceleration injuries involving the great vessels.^2^ Malignant effusions are often blood-stained, but not associated with myocardial wall rupture.

**Figure 2:**
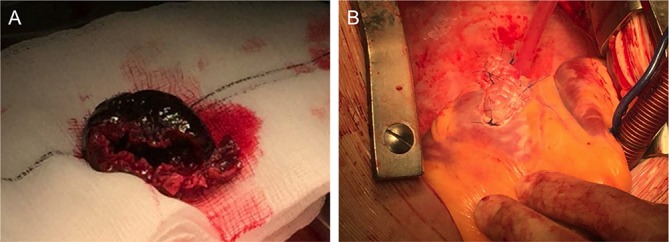
Wide-necked inferolateral wall left ventricular pseudoaneurysm and thrombus resection (A) and repair at the site of mature scar formation (B).

Surgical evacuation and patching of the pseudoaneurysm was performed to relieve cardiac tamponade (see Figure 2 and online supplementary movie). The infarcted tissue was mature with macroscopic scarring. Contained rupture is rare following myocardial infarction and confers a poor prognosis (75% inhospital mortality).^3^ It is typically a late complication of myocardial infarction, diagnosed within 6 months following episodes of recurrent chest pain. Inferolateral pseudoaneurysms are more frequently contained by pericardial adhesions, thus preventing rapid deterioration.^4^ The patient recollected an episode of ischaemic-sounding chest pain 8 months ago, for which he did not seek medical attention (see online supplementary figure). Left ventricular pseudoaneurysm formation was a complication of transmural infarction due to right coronary artery occlusion.

10.1136/heartjnl-2016-310923.supp1 Supplementary movie

10.1136/heartjnl-2016-310923.supp2 Supplementary Figure
