# Supplementary figures and images for: A 49-year-old with chest pain and collapse
Source: Heart. 2017 Mar 11;103(12):968. doi: 10.1136/heartjnl-2016-310923 (PMC5566090; doi:10.1136/heartjnl-2016-310923)

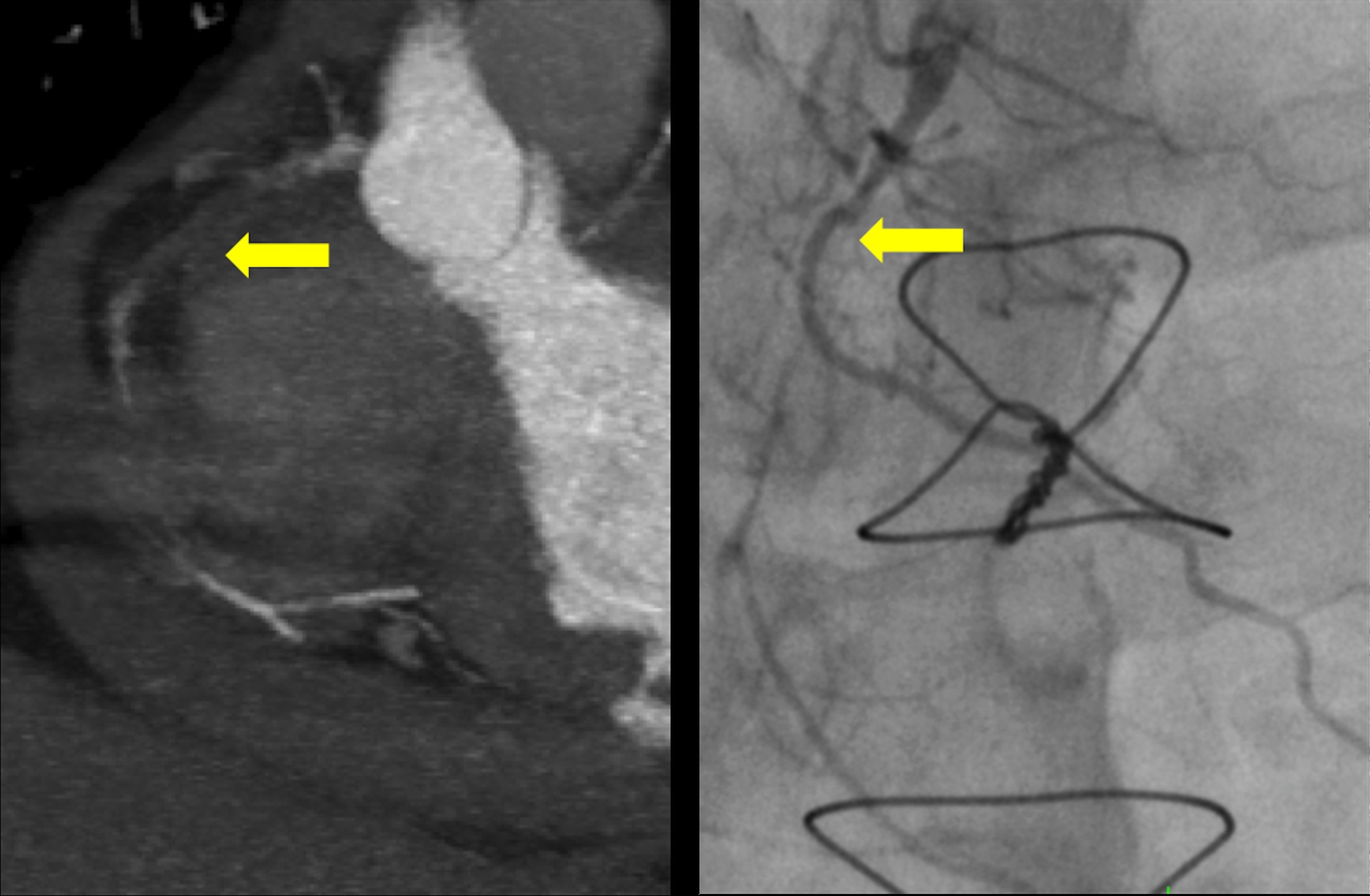

Supplement: Supplementary Figure [file heartjnl-2016-310923supp002.jpg]
